# Children’s and Adults’ Sensitivity to Gricean Maxims and to the Maximize Presupposition Principle

**DOI:** 10.3389/fpsyg.2021.624628

**Published:** 2021-03-03

**Authors:** Francesca Panzeri, Francesca Foppolo

**Affiliations:** Department of Psychology, University of Milano-Bicocca, Milan, Italy

**Keywords:** maxims of conversation, acquisition of pragmatics, experimental pragmatics, maximize presuppositions, maxim of manner, pragmatic tolerance

## Abstract

Up to age 5, children are known to experience difficulties in the derivation of implicitly conveyed content, sticking to literally true, even if underinformative, interpretation of sentences. The computation of implicated meanings is connected to the (apparent or manifest) violation of Gricean conversational maxims. We present a study that tests unmotivated violations of the maxims of Quantity, Relevance, and Manner and of the Maximize Presupposition principle, with a Truth Value Judgment task with three options of response. We tested pre-schoolers and school-aged children, with adults as controls, to verify at which age these pragmatic rules are recognized and to see whether there is a difference among these tenets. We found an evolutionary trend and that, in all age groups, violations of the maxims of Quantity and of Relation are sanctioned to a higher degree compared to infringements of the Maim of Manner and of the Maximize Presupposition principle. We conjecture that this relates to the effects that the violation of a certain maxim or principle has on the goals of the exchange: listeners are less tolerant with statements that transmit inaccurate or incomplete information, while being more tolerant with those that still permit to understand what has happened.

## Introduction

In his seminal work, Grice (1975) proposed an account of how speakers can communicate more than what they literally say based on the assumption that rational interlocutors collaborate to reach a common objective and that the most efficient way to accomplish this goal is to follow the maxims of conversation. These maxims regulate both the content of what is said—that has to be true (maxim of Quality), enough informative (maxim of Quantity), and relevant (maxim of Relation)—and its form: statements are required to be clear, unambiguous, concise, and with the events reported orderly (maxim of Manner). In interpreting speakers’ remarks, then, the hearers assume that these general principles are obeyed; faced with apparent or manifest violations of these maxims, they will integrate new assumptions to save the cooperativity of the speakers. These implicit propositions, the implicatures, integrate what a speaker has said and permit the recognition of speakers’ communicative intent. For instance, a speaker saying “Leo drew a dog in his notebook” will be normally taken to implicate that the dog was the only thing Leo drew, since if he had drawn something else (also a cat, for instance), a cooperative speaker who complies with the maxim of Quantity should have mentioned it. Or, if one utters “Lawyers are sharks,” a statement that is obviously false if taken at face value, interlocutors will reinterpret it as a metaphoric comment under the assumption that the speaker is cooperative at a deeper level. In other words, within the pragmatic perspective that views the recognition of the speaker meaning as an inferential process that starts from what a speaker has said and integrates it with additional assumptions, the conversational maxims play a key role in the derivation of these implicitly transmitted propositions, the implicatures. The apparent or manifest violations of the maxims, as in the cases discussed above, trigger the derivation of additional premises to save the appropriateness of the remark.

Numerous studies found that children experience many difficulties in the correct detection of the speaker intended meaning: they tend to stick to a literal interpretation of what was said without integrating the implicitly conveyed assumptions. Children, for instance, struggle with instances of figurative language for a long period: the correct interpretation of metaphors and ironic comments is a late developing skill (see Winner, 1997, for a review). Moreover, children up to at least 5 years of age do not compute the generalized conversational implicatures that ensue from the assumption that the speaker is obeying the maxim of Quantity: when presented with a scenario in which, for instance, Leo ate all the five cookies that were on a plate, if they are asked to evaluate the appropriateness of a sentence such as “Leo ate *some of* the cookies,” preschoolers accept it, whereas adults reject it. The rejection of that sentence is couched on the derivation of the Quantity implicature: the utterance of “Leo ate some of the cookies” implicates that stronger statements (such as “Leo ate all of the cookies”) are not true because the maxim of Quantity dictates to utter the most informative true statement.

The fact that younger children tend not to derive implicatures has received a lot of attention, and different proposals have been put forth to account for this non-adult-like behavior. Within a Relevance theory perspective that assumes that the derivation of implicit content is guided by the balance between the costs required by the activation of the inferential process and the cognitive gains that permit the strengthening of what is said, children might not be able to optimize this balance yet: they would be satisfied with a literal, unenriched interpretation of the statement because they do not realize that the cost of deriving implicit additional assumptions would result in a strengthened, and thus more informative, interpretation (Pouscoulous et al., 2007). Other scholars suggest that children might encounter difficulties in the identification of the salient alternatives that are necessary to trigger the inferential mechanism (Skordos and Papafragou, 2016): in the Quantity implicatures discussed above, children might not be able to understand that the statement “Leo drew a dog” or “Leo ate some of the cookies” is not felicitous in a scenario in which Leo drew both a dog and a cat or in which Leo ate all of the cookies because they are not able to retrieve the relevant alternatives that the speaker should have uttered to adhere to the maxim of Quantity. Finally, children might simply be more tolerant than adults with respect to pragmatically inappropriate statements: presented with a statement that omits important details to describe a given situation (e.g., “Leo drew a dog” when in fact he drew both a dog and a cat), children might accept it, being satisfied with the semantic truth of the statement and not sanctioning its pragmatic inappropriateness. To test this hypothesis, Katsos and Bishop (2011) proposed a Truth Value Judgment (TVJ) task, in which participants are presented with a statement that is true but underinformative in a given scenario in two different versions. In the first version, participants had to evaluate a puppet’s statements using two options of response; in line with previous findings, children accepted, and adults rejected, true but underinformative statements. In a second version, though, participants were asked to reward the puppet with three different sized strawberries: in this case, children did not differ from adults and preferred the middle-sized reward to judge true but pragmatically inappropriate (i.e., underinformative) statements. Katsos and Bishop (2011) thus concluded that children are indeed sensitive to violations of pragmatic appropriateness, but they do not sanction it as adults do.

The studies discussed above focused on children’s failure to derive implicatures under the assumption that the speaker is complying with the maxim of Quantity and hypothesized that children might exhibit a non-adult-like behavior, accepting true but underinformative statements, either because they are simply more tolerant with respect to pragmatic inappropriateness or because they experience difficulties in the inferential process, in particular in the identification of the relevant, more informative, statements that a cooperative speaker should have chosen. A different but related question is whether this pragmatic immaturity is specific to the maxim of Quantity or whether it involves other Gricean conversational maxims.

This question has been tackled by Surian et al. (1996): they designed a Felicity Judgment (FJ) task, the Conversational Violations Test (CVT), in which children were presented with two puppets that answered to various questions, and the child’s task was to identify who was giving silly answers: the target puppy was the one that was violating one of the maxims of conversation. In particular, besides testing violations of the Principle of Politeness (with one puppet providing rude remarks), the CVT tested violations of the maxim of Quality (with one puppet providing an impossible-to-be-true answer, e.g., “I live on the moon”), of the maxim of Relation (with a puppet providing completely irrelevant answers, such as “My trousers were blue,” to the question “What did you do on holiday?”), and of the two submaxims of Quantity. The first submaxim (Quantity I) prescribes to provide as much information as is required; in the CVT, for instance, when asked “What did you receive for your birthday?” the silly puppet replied “A present,” a clearly underinformative answer. The second submaxim of Quantity (Quantity II), on the other hand, invites speakers not to provide more information than what is required. Surian et al. (1996) exemplified violations of this submaxim, which they labeled “Avoid redundant information,” with statements such as “[For breakfast, I had] a hard-boiled egg cooked in hot water in a sauce pan.”

We actually believe that these items exemplify violations of the Be brief submaxim of Manner and not of Quantity II. Grice (1975) admitted that the second submaxim of Quantity was “disputable” and discussed, with many cautionary remarks, a case in which it gets violated: A asks B whether p is the case, and B “volunteers not only the information that, but information to the effect that it is certain that p, and that the evidence for being the case that p is so-and-so and such-and-such” (Grice, 1975, p. 52). He then discusses a violation of Be brief: the utterance of “produced a series of sounds that corresponded closely with the score of ‘Home sweet home,”’ instead of the more concise “sang ‘Home sweet home”’ (Grice, 1975, p. 55). The items tested in the CVT constitute, in our view, unnecessary lengthy descriptions (a “rigmarole,” in Grice’s words) that do not add any other information compared to more concise expressions, and we therefore consider them as violations of Be brief and not of Quantity II.

The CVT has been used to test atypical populations (children with autism spectrum disorders and with specific language impairment, Surian et al., 1996; hearing-impaired children, Surian et al., 2010) and bilingual children (Siegal et al., 2009, 2010), with typically developing (TD) monolingual children serving solely as the control group. Indeed, the evolutionary trajectory of TD children in the mastering of Gricean maxims has not been attested yet, except for one study that used a revised CVT in Japanese-speaking children, aged 4–6 years (Okanda et al., 2015). In this study, moreover, Okanda et al. (2015) highlighted how the CVT did not test violations of the maxim of Manner, and they thus decided to test cases in which the question was “Which do you like, tea or milk?” and the puppets answered either “I like milk” or “Maybe tea or maybe milk,” an answer that they consider a violation of the submaxim Avoid ambiguity.

Besides the maxims of conversation, another pragmatic principle has been proposed: the Maximize Presupposition principle (Heim, 1991). Analogously to what happens with conversational implicatures, also this principle requires the evaluation of semantically equivalent alternatives that differ in their pragmatic appropriateness. Following Heim’s example, the statement “*A* (biological) father of the victim arrived at the scene” sounds anomalous compared to “*The* (biological) father of the victim arrived at the scene.” The determiner phrases “a D” and “the D” differ in that the latter presupposes the existence of a unique object that satisfies the description D. Since every person has a unique biological father, this presupposition of (existence and) uniqueness is indeed satisfied. Maximize Presupposition accounts for the infelicity of using the determiner “a” instead of “the” by stating that it is more pragmatically appropriate to use the alternative that activates presuppositional requirements that are satisfied in the context.

Sauerland (2008) explicitly drew a parallelism between the application of this Maximize Presupposition principle and the derivation of (Quantity I) implicatures: in both cases, there are alternative statements that differ in “strength” because one element is more informative or activates presuppositions; if a speaker utters the weaker statement, an implicature or implicated presupposition can be drawn that the stronger statement does not hold. Or, conversely, the utterance of the weaker element when the stronger one could have been used is pragmatically inappropriate.

To test whether children are sensitive to the Maximize Presupposition principle, Yatsushiro (2008) tested children aged 6–9 years and adults by means of an FJ task: participants were presented, for instance, with a picture of a single girl playing soccer, and they were asked to indicate which of the statements “The girl is playing soccer” and “Every girl is playing soccer” best described the situation. Despite the fact that the second statement is semantically true (under its logical reading, it simply requires that all the individuals who are girls in a given context are playing soccer), the statement with the definite description *the girl* should be preferred, given that the uniqueness presupposition associated to *the* is satisfied. Yatsushiro (2008) found an evolutionary trend, with 6-year-olds performing worse (albeit showing an accuracy above 70%) than 7-year-olds. At 8 years of age, though, children were adult-like, with an accuracy above 90%. FJ tasks such as the CVT and the one employed by Yatsushiro (2008) tap the ability of children to recognize which one of the two presented statements violates one of the Gricean maxims or complies with the Maximize Presupposition principle. Nevertheless, choosing the correct answer in an FJ task does not necessarily indicate that the child is aware that an answer that violates the Maximize Presupposition principle or a maxim is pragmatically inadequate: when presented with two statements, one that violates and one that complies with pragmatic principles, the child could be simply identifying the (more) appropriate answer. Indeed, Foppolo et al. (2012) tested two groups of monolingual 5-year-old children who failed to derive scalar implicatures in a classical TVJT with the CVT adopted from Surian et al. (1996) and with an FJ task in which children had to compare an underinformative vs. an optimal alternative description of a situation. They found that, despite accepting underinformative statements violating the maxim of Quantity in the TVJ task, most of the children performed at ceiling in the CVT and in the FJ task, suggesting that these tasks might overestimate children’s pragmatic competence.

To verify whether children appreciate Gricean maxims and the Maximize Presupposition principle, that is, whether they recognize when a statement is not appropriate in a given situation, we should resort to tasks such as the TVJ task, where the child is asked to evaluate the felicity of single statements against a scenario. Only if children correctly reject utterances that do not conform to Gricean maxims and that do not follow the Maximize Presupposition principle we can safely conclude that they are aware of these pragmatic principles.

Building on previous work, we designed a task that aims at testing children’s sensitivity to pragmatic principles using a ternary TVJ task in which participants have to judge the appropriateness of a single statement in a given scenario, evaluating it with three options (bronze, silver, or gold medal). On the one hand, this should enhance children’s performance compared to binary tasks, as already observed by Katsos and Bishop (2011); on the other, differently from FJ tasks, in this task, children have to evaluate one single statement at a time, without being provided with an alternative, i.e., a pragmatically appropriate description, rendering the task more apt to capture children’s real competence. Our main goal was to verify at which age children realize that, if a speaker violates a conversational tenet (a Gricean maxim or the Maximize Presupposition principle) for no clear purpose, then the resulting utterance is infelicitous. Target items comprise violations of the Maximize Presupposition principle, of the maxim of Relation, of Quantity I, and of two submaxims of Manner, Be brief and Be orderly. Control items constitute literally true and false statements: notice that false statements can also be seen as unmotivated violations of the maxim of Quality.

Since Grice himself stated that “the observance of some of these maxims is a matter of less urgency that is the observance of others” (Grice, 1975, p. 46) and that, in particular, a speaker who uses undue prolixity (violating the submaxim of Manner Be brief) is more cooperative than one who lies (violating Quality), we may expect differences in the rejection rates across the maxims. Another goal of the present study, then, is to compare the relative impact of maxims’ violations and the consequent sanctioning of those violations across different maxims and age groups. In particular, since many studies employing a TVT task found that children up to 5 years of age tend to accept statements that violate Quantity I, not deriving scalar implicatures, we aimed at using the same task to verify whether unmotivated violations of other maxims are sanctioned at the same level.

## Materials and Methods

### Participants

We tested a total of 163 Italian monolingual TD children, 45 (22 F) were preschoolers, with a mean age of 5 years and 2 months (age range: 3.7–6.2), and 118 (68 F) were school-aged children enrolled in the first 3 years of primary school (40 first graders; 30 second graders; 48 third graders), with a mean age of 7.5 (age range 6–9). A group of 36 adults (18 F, mean age 36 years) served as control.

### Materials and Procedure

**FIGURE 1:**
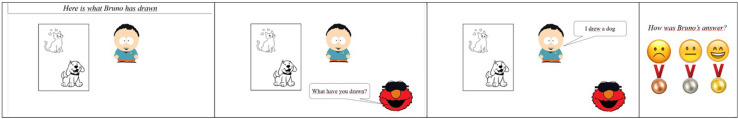
Exemplification of a critical trial involving the violation of the maxim of Quantity.

In the task, children were asked to evaluate the appropriateness of statements uttered by a boy, Bruno, who answers to the questions of a puppet, Elm. In the warm-up sessions, children were introduced to the two characters: they were told that Bruno is a boy who does many things and that Elm is very curious to know what happens, but he is blindfolded, and therefore he poses a lot of questions to Bruno. Children were warned that Bruno always answers Elm’s questions, but sometimes Bruno’s answers are wrong, or at least not completely adequate: in those cases, children should warn Elm, and tell him what has really happened. For each item, participants were first provided with a scenario about what Bruno did, then Elm comes in and poses a question to Bruno, and he answers with a fully informative, underinformative, or false statement about what he did. The child task was to judge the appropriateness of Bruno’s statement relative to the given context. Following Katsos and Bishop (2011), they had to select one of three options: gold medal/smiley face for really appropriate answers, bronze medal/sad face for completely wrong ones, and silver medal/blank face for so-and-so answers (i.e., true but somehow misleading descriptions of the context). For example, they were presented with the scenario in Figure 1 (in which Bruno drew a cat and a dog), and they were told, “Here is what Bruno has drawn,” then Elm comes in and poses his question to Bruno, “What have you drawn?” Bruno answers, “I drew a dog,” thus violating the maxim of Quantity. Finally, the child is asked to judge Bruno’s statement by selecting the bronze, silver, or golden medal.

The task comprises 24 items: 12 critical items and 12 control sentences. The critical items constitute unmotivated violations of the maxim of Quantity (four items), Relation (two items), and Manner (four items) and of the Maximize Presupposition principle (two items). As for the Quantity maxim, the critical statements are underinformative with respect to the given context, thus violating Quantity I (“Make your contribution as informative as is required”), either because Bruno mentions only one conjunct instead of two (as in Figure 1) or because he mentions the superordinate term instead of the basic one (e.g., instead of answering “I’ve eaten chicken,” he says “I’ve eaten food”). The two items that involve unmotivated violations of the maxim of Relation constitute irrelevant answers to a question: for instance, participants are shown Bruno’s favorite shirt (red, with an image of a monkey) that Bruno describes as a shirt “that has two sleeves and a hole for the head.” Infringements of the maxim of Manner are tested with two items that violate the submaxim Be orderly (e.g., “I went to the bed and I brushed my teeth”) and two items that violate the submaxim Be brief (e.g., Bruno said that his snack was “A fruit with yellow peel and that monkeys really like,” instead of simply saying “banana”). As discussed in the *Introduction*, these items are lengthy descriptions that do not add any information to more concise terms, and we thus consider them as violations of Be brief, whereas in the CVT, they were viewed as involving Quantity II.

Notwithstanding, since it is admittedly disputable what maxim is involved, in the *Discussion* section, we take into account also the hypothesis that the second submaxim of Quantity is implicated.

Finally, two critical items were violations of the Maximize Presupposition principle, in which the indefinite determiner *a* was used instead of the stronger presuppositional trigger *the* (e.g., “A sun is setting”). The control statements were eight clearly true statements and four clearly false. Notice that the false statements can also be viewed as unmotivated violations of the maxim of Quality, in particular, of the first submaxim “Do not say what you believe to be false.” Since the critical items that constitute violations of Be brief were long statements, some of the control items were lengthy descriptions, some of which were true (for instance, “I like to have tea with lemon and sugar”) and some false (“On the desk, there is a sheet of paper, colored pencils, and a book”—when there were watercolors instead of a book).

In an initial warm-up session, participants were familiarized with the task. This session comprised a clearly true and a clearly false statement (to be rewarded with gold and bronze medal, respectively) and an instance of a so-and-so type of answer: participants were first shown an image of Bruno’s favorite pizza (with sausages and French fries), then Elm asked Bruno how he liked to eat pizza and he answered that he likes the pizza on a plate.

The task was implemented on Microsoft PowerPoint, with all the statements prerecorded and presented auditorily to children or presented in written form on the screen (for adults). A researcher annotated participants’ answers on a sheet of paper. Children were tested in a quiet room of their schools after parents signed a consent form. All of them completed the task. Adult participants were recruited on a voluntary basis.

## Results

The distribution (in percentages) of children’s and adults’ responses on the ternary scale for True and False controls and critical items is summarized in Table 1.

**TABLE 1:** Distribution of response types (bronze/silver/gold medals) in the experimental target conditions (violations of maxims and of Maximize Presupposition) and in the True/False control conditions.

	**Preschool children**			**Primary-school children**			**Adults**		
	**Bronze medal**	**Silver medal**	**Gold medal**	**Bronze medal**	**Silver medal**	**Gold medal**	**Bronze medal**	**Silver medal**	**Gold medal**
True	0%	2.2%	97.8%	0.5%	6.4%	93.1%	0.3%	4.5%	95.1%
False	66.1%	25.6%	8.3%	63.3%	34.1%	2.5%	71.5%	27.8%	0.7%
Target	15.7%	24.4%	59.8%	11.3%	51.1%	37.6%	9.5%	69.4%	21.1%

All participants responded with the fully positive option in True controls (>95% of gold medals in all groups); in general, they also rejected False controls above 90% of the time by selecting either the bronze or the silver medal. Specifically, they selected the bronze medal above 60% of the time. The high percentage of silver medals for control statements that were literally false was somehow unexpected; however, this was mainly due to the items that constituted partially true descriptions of the situation, like the long statement discussed before, in which two out of three objects that were indeed present on the desk were mentioned. In the target conditions, i.e., those that involved a violation of pragmatic principles, the older children and adults selected the middle option in the majority of the cases, as expected, while the younger children chose the gold medal in the majority of the cases, although about one fourth of the time, they selected the silver medal. All groups tended to be more polarized in their answers in the control conditions compared to the target conditions.

1To evaluate the pattern of responses between target and control conditions, and among age groups, we implemented a mixed-effects ordinal regression model with a logit link function using the *clmm*() function in the *ordinal* package (Christensen, 2018). This is a statistical model specifically designed to treat ordinal-dependent measures that cannot be assumed to represent an interval scale, as it is the case with the ternary option used in our study. The maximal model that converged included Condition (control vs. target) and age Group (preschoolers, primary-school children, and adults) as fixed effects, and their interactions, as well as participants and items as random intercepts. We used dummy coding for Condition and age Group so that control items and primary-school children served as the baselines in the contrasts. The model (Table 2) reported a difference in the distribution of medals between the younger and the older children, while no difference was revealed between primary-school children and adults. This suggests a developmental trend that was further explored in a second model. No fully significant interactions were reported. However, we have to take this result with some caution due to the relatively small number of data points considered in the analyses.

**TABLE 2:** Output of the model with type of Medal as the dependent variable, Condition and age Group as independent variables (dummy coded with control and primary-school children as baselines), and subjects and items as random intercepts.

	**Estimate**	**Std. Err.**	***z*-value**	***p*-value**
Cond (target vs. control)	−1.386	1.203	−1.152	0.249
Group (adults vs. primary-school children)	−0.201	0.252	−0.798	0.425
Group (preschool vs. primary-school children)	0.536	0.239	2.245	0.025
Cond: Group (adult)	−0.388	0.217	−1.783	0.075
Cond: Group (preschool)	0.312	0.214	1.460	0.144

We then ran a second model in which we contrasted the experimental items (settled as the baseline) with True and False controls considered separately in the three age groups. This model revealed a significant difference of primary-school children both with the preschool children and adults, as well as significant interactions between item Type and age Group (Table 3). The interaction between Type and age Group was significant in the comparison between the younger and the older children in the case of False controls (*p* = 0.002), while it only approached significance in the case of True controls (*p* = 0.057), again suggesting a developmental trend in children’s ability to conform to the task and to detect the violations of conversational maxims, which improved with age. A significant interaction of Type and Group was also revealed in the case of True controls between primary-school children and adults, suggesting that the older children, despite being more pragmatically mature than the younger children, were not fully adult-like yet in the treatment of statements that violated a maxim, accepting them at a higher rate compared to adults.

**TABLE 3:** Output of the model with type of Medal as the dependent variable, Type (target items vs. False/True controls) and age Group as independent variables (with primary-school children as the baseline), and subjects and items as random intercepts.

	**Estimate**	**Std. Err.**	***z*-value**	***p*-value**
Type (false vs. target)	−3741	0.698	−5.361	<0.001
Type (true vs. target)	3.747	0.566	6.619	<0.001
Group (adults vs. primary-school children)	−0.589	0.206	−2.861	0.004
Group (preschool vs. primary-school children)	0.859	0.198	4.343	<0.001
Type (false): Group (adult)	0.021	0.273	079	0.937
Type (true): Group (adult)	0.930	0.333	2.792	0.005
Type (false): Group (preschool children)	−0.817	0.257	−3.175	0.002
Type (true): Group (preschool children)	0.794	0.416	1.907	0.057

**FIGURE 2:**
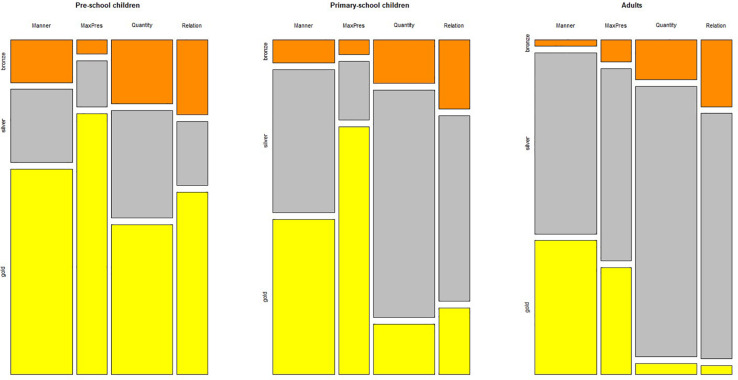
Distribution of response options across pragmatic violations and age groups.

To investigate whether a different pattern was revealed across different types of violations of maxims, and across different age groups, we focused on the target items only. The distribution of the response options across maxims violations and age groups is plotted in Figure 2; the mean percentages are reported in Table 4.

**TABLE 4:** Distribution of medals (in percentages) for all the types of violations (with the submaxims of Manner, Be brief and Be orderly, provided separately) and all age groups.

	**Preschool children**			**Primary-school children**			**Adults**		
	**Bronze medal**	**Silver medal**	**Gold medal**	**Bronze medal**	**Silver medal**	**Gold medal**	**Bronze medal**	**Silver medal**	**Gold medal**
MaxPres	4.4%	14.4%	81.1%	4.7%	18.2%	77.1%	6.9%	59.7%	33.3%
Manner: Be brief	16.7%	23.3%	60%	2.1%	53%	45%	2.8%	66.7%	30.6%
Manner: Be orderly	10%	22.2%	67.8%	12.3%	36%	51.7%	1.4%	45.8%	52.8%
Quantity I	20%	33.3%	46.7%	13.6%	70.8%	15.7%	12.5%	84.0%	3.5%
Relation	23.3%	20.0%	56.7%	21.6%	57.6%	20.8%	20.8%	76.4%	2.8%

We ran four separate models to compare the different maxims’ violations, as well as the interaction for each of the age groups, by changing the level of the variable to serve as the baseline for each of the contrasts. For better readability, we discuss the main results in the paper and refer to the Appendix for the full model outputs (Appendix Tables A–D). In general, all maxim violations differ from the others (all *p* < 0.001) except for the maxims of Quantity and Relation, which do not differ (*p* = 0.783). As for a general effect of age Group, primary-school children differ from preschool children in detecting the violations of the maxims of Manner, Quantity, and Relation (all *p*s < 0.05). In these conditions, the preschool children tended to be more tolerant than the older children, selecting the gold medal most of the time, compared to the older children, who selected the silver medal in most of the cases. Comparing primary-school children with adults, the only significant difference is in the detection of the violation of the Maximize Presupposition principle: in this case, the primary-school children tended to be significantly more tolerant than adults, selecting the gold medal at a higher rate compared to adults (*p* < 0.001). This is also captured by the three significant interactions of Group (adults vs. primary-school children) and type of Violation in the contrasts in which Maximize Presupposition is set as the baseline (all *p*s < 0.001).

The maxim of Manner was tested with two items that violated the submaxim Be brief and two items that violated the submaxim Be orderly. As discussed above, unnecessary lengthy descriptions were viewed by Surian et al. (1996) as involving the second submaxim of Quantity, whereas we considered them as related to Be brief. It is therefore relevant to further inspect these items and in particular to verify whether they are treated like Be orderly or like Quantity I violations. In Table 4, we provide the distribution of medals for all pragmatic violations, splitting the maxim of Manner in its two submaxims, Be brief and Be orderly, in all age groups.

We ran three additional models, one for each age group separately, in which we considered submaxims as the independent variable, setting Be brief as the baseline. In this way, we could evaluate the comparison between violations of the two submaxims of Manner, as well as the difference, if any, between Be brief and the first submaxim of Quantity in each age group. In all age groups, no difference was revealed in the distribution of medals between Be brief and Be orderly (all *p*s > 0.05). As for the comparison between Be brief and the first submaxim of Quantity, the models revealed a significant difference in adults and primary-school children (all *p*s < 0.001); in preschool children, instead, no difference was revealed between any of these conditions, as in all cases, young children seem to equally tolerate such violations (*p* = 0.258, see Appendix Tables E–G for the full output of the models).

## Discussion

In this study, we aimed at assessing whether children are sensitive to violations of pragmatic principles, testing unmotivated violations of the Gricean maxims of Quantity, Relation, and Manner, and of the Maximize Presupposition principle (violations of the maxim of Quality were controlled by means of False controls). In particular, we wanted to determine whether children’s pragmatic competence improves with age and whether there is a difference in the sanctioning of violations of different maxims and of the Maximize Presupposition principle. We used a TVJ task with three options of response, since Katsos and Bishop (2011) found that a binary task could blur children’s ability to recognize the infelicity of underinformative sentences. All the critical items constituted literally true statements that were pragmatically inappropriate in the given scenario because they were infringing one of the pragmatic tenets. The expected answer, then, was a rejection of these remarks, assigning to Bruno, the speaker, the silver or the bronze medal.

Taking into account the contrast between control and target items, the first comparison across the age groups revealed an evolutionary trend, with primary-school children differing from the younger children in their choices, but not from adults. In the case of a violation of a maxim, the younger children selected the gold medal almost 60% of the time, while about 37 and 21% of the older children and adults did so, respectively, showing that pragmatic violations are sanctioned more with age. In fact, the majority of the older children and the adults sanctioned the infelicitous statement by selecting the silver or the bronze medal, although the older children overall accepted the violations of the maxims more than the adults, as emerged in the second model. Notice that, following Katsos and Bishop (2011), the TVJ task contemplated three options of response; still for younger children, the gold medal constitutes the preferred choice for all pragmatic violations.

Focusing on the pragmatic principles, we found a different degree of tolerance depending on the kind of violations. First of all, the items in which the maxims of Quantity or Relation were violated behaved similarly and were significantly less accepted than all the other violations across all age groups. In these cases, adults rejected the statements 97% of the time. Similarly, these violations were the most sanctioned by children. Related to this point, it is worth mentioning that all participants sanctioned False controls, which are indeed violations of the maxim of Quality, above 90% of the time.

Second, a difference was revealed both in adults and primary-school children between their reaction to the violation of the submaxim of Be brief (that in the CVT was regarded as a violation of Quantity II) and of Quantity I, speaking in favor of a different treatment of these two types of violations.

Third, it is interesting to notice that adults behaved somehow unexpectedly in the case of violations of the maxim of Manner and of the Maximize Presupposition principle. As is evident from Figure 2, they often chose to assign the gold medal to items that infringe these tenets, accepting unnecessary long answers or statements in which events are reported in reverse order (violations of the maxim of Manner received a gold medal 42% of the time) and in which the indefinite determiner *a* is used instead of the stronger, presuppositional triggering determiner *the* (violations of the Maximize Presupposition principle received a gold medal 33% of the time). Indeed, children accepted statements that violated the Maximize Presupposition principle even more than the adults, suggesting that this type of violation is fully accepted in most cases (77 and 81% of gold medals for older and younger children, respectively).

We will discuss the implications of these findings in turn. First of all, the fact that violations of Quantity and Relation differ from all the others highlights the different statuses of pragmatic principles. As already alluded to, Grice himself suggested that the observance of the maxims could be a matter of more or less “urgency.” The maxim of Quality has always been considered to be special, since its violation does not to lead to simply “an inferior kind of information; it just is not information” (Grice, 1989, p. 371). Our results corroborate this, showing a high rate of sanctioning of False controls by adults and children. In our study, though, we also found evidence for a clear differentiation also among the maxims of Quantity and Relation, on the one hand, and of the maxim of Manner and of the Maximize Presupposition principle on the other hand: while adults (and children) always sanction the maxims of Quantity and Relation, they are more tolerant with violations of the maxim of Manner and the Maximize Presupposition principle, accepting them to a higher degree.

To account for this difference, we can notice how violations of these maxims result in different outcomes: if a speaker violates the maxim of Quantity or Relation, the hearer cannot really understand what has happened. In our example, for instance, when Bruno says that he drew a dog, the blindfolded Elm will come to believe that the dog was the only thing that Bruno drew, under the assumption that Bruno is cooperative, and he will thus form an incorrect belief about the situation, since Bruno also drew a cat. Again, when Bruno answers that he ate food for lunch or that his favorite shirt has two sleeves and a hole for the head, Elm does not have the necessary and relevant information that would permit to understand what Bruno ate (a chicken) and what his favorite shirt looks like (red, with a monkey on it). The statements that infringe the maxims of Quantity (at least the first submaxim, Be enough informative) and of Relation, in other words, transmit inaccurate or incomplete information. The two submaxims of Manner Be brief and Be orderly and the Maximize Presupposition principle, on the other hand, regulate the form, and not the content, of the statements. If speakers provide unnecessary long descriptions, or if they report the events in a reverse order, or if they fail to use the stronger, presuppositions triggering expressions, they still transmit a piece of information that enables interlocutors to understand what has really happened. Thus, the rigmarole “a fruit with yellow peel and that monkeys really like” enables interlocutors to understand that the speaker is referring to a banana; hearing “I went to the bed and I brushed my teeth” permits Elm to understand which events took place, even if they are mentioned in the wrong order; similarly, the anomalous statement “A sun is setting” correctly depicts the situation of a sunset. We therefore hypothesize that the interlocutors sanction infringements of the maxims in different ways, rejecting those that transmit inaccurate or incomplete information and being more tolerant with those that still permit to understand what has happened.

To further speculate on this finding, we discuss an interesting parallelism that comes from the literature on referential expressions. Engelhardt et al. (2006) tackled the question whether adults are sensitive to the first (Be enough informative) and to the second (Do not be too informative) submaxim of Quantity. They focused on descriptions of a target referent that were underinformative (e.g., “the apple” when two different apples were present) or overinformative (e.g., “the apple on the towel” when only one apple was present) in tasks that required to produce or comprehend commands such as “Put the apple (on the towel) in the box.” They found an asymmetry: referential expressions that did not provide enough information for the correct identification of the target (violating Quantity I) were never produced and were penalized in comprehension; descriptions that provided more information than what was strictly required to identify the referent (violating Quantity II) were spontaneously produced 30% of the time and were not rated significantly lower than optimally concise descriptions. Quite interestingly, though, in a third eye-tracking experiment, they found that overinformative descriptions did cause momentary confusion, indicating that unnecessary modifications are costlier to process. Even if Davies and Katsos (2013) argued that the “only moderately Gricean” behavior of participants in Engelhardt et al. (2006) studies might be due to methodological confounds linked to the complexity and visual salience of the array of stimuli, it is interesting to comment on these data.

Analogously to what was observed by Engelhardt et al. (2006), we found that violations of Quantity I are always sanctioned; following their explanation, we argue that this might be so because a lack of necessary information “can compromise communication, as an under-described utterance will not permit a listener to identify the correct referent from a set” (Engelhardt et al., 2006, p. 563); being overinformative, on the other hand, may cause a temporal ambiguity but does not block the identification of the referent and, for this reason, might be less sanctioned and even be spontaneously produced.

As already discussed in the *Introduction*, it is “disputable” whether unnecessary long descriptions constitute violations of Quantity II or of Be brief. What these kinds of “harmless” violations of the maxims have in common is that they do not lead to a communication failure, as much as violations of Quantity I (and of Relation) would do, and they are therefore less sanctioned. We argue that violations of Quantity II, “on the assumption that the existence of such a maxim should be admitted” (Grice, 1975, p. 52), are analogous to infringements of the submaxims of Manner Be brief and Be orderly and of the Maximize Presupposition principle.

With respect to the final point listed above, our results for the Maximize Presupposition principle limit the scope of Sauerland (2008)’s claims: even if Maximize Presupposition and Quantity I behave similarly, in that they both demand the choice of the stronger alternative statement, they also differ because violations of the former are viewed as less detrimental compared to statements that do not transmit enough information. In fact, the extremely low rejection of statements that violate Maximize Presupposition contrast with the results in Yatsushiro (2008), who found that children already reached 70% accuracy at age 6, even if they performed worse than the 7-year-olds, and only performed adult-like at age 8. In our study, we found that primary-school children, aged 6–9, rewarded the violations of the Maximize Presupposition principle with a gold medal 77% of the time. This difference might be due to the type of Maximize Presupposition violation presented in the two studies or to the type of task used. We presented statements with the indefinite determiner *a* instead of the more appropriate *the*, whereas Yatsushiro (2008) contrasted the definite description with the determiner *every*. Moreover, we used a TVJ task with ternary options, whereas Yatsushiro (2008) employed an FJ task. As already discussed, FJ tasks might overestimate children’s actual pragmatic competence (see also Foppolo et al., 2021): choosing the more appropriate alternative does not necessarily mean that when presented with the pragmatic infelicitous statement, children would reject it, as already discussed by Foppolo et al. (2012).

To conclude, the results of the present study offer potentially interesting lines of research. On the one hand, the question whether interlocutors are only “moderately” (Engelhardt et al., 2006) or “fully” (Davies and Katsos, 2013) Gricean remains open and should be further explored. We found that adult participants always penalized statements that violated the maxims of Quality, Quantity I, and Relation, whereas they were more tolerant with regard to infringements of the two submaxims of Manner Be brief and Be orderly. Even if this observation is in our view coherent with Grice’s own view, since he explicitly recognized that the observation of maxims is indeed a matter of more or less urgency, it is worth exploring the conditions under which interlocutors might attribute more or less importance to the maxims of conversation. As already alluded to, Davies and Katsos (2013) argued that the apparent tolerance of overinformative statements in Engelhardt et al. (2006) might be imputed to contextual factors: being presented with a complex array of objects, participants might prefer overdescriptions to more concise expressions to avoid ambiguity. Still, as Engelhardt (2013) highlighted, the conditions that might push interlocutors to prefer avoiding possible ambiguity instead of choosing optimally concise expressions need to be further explored. Also, consider that the visual display in our task was extremely simple, and the excessively verbose description was referred to a single object.

Another factor that might have sharpened the difference between the violations of Quantity I and Relation, on the one hand, and of violations of Be brief, Be orderly, and Maximize Presupposition, on the other, is the fact that in our task, participants had to evaluate a single speaker, Bruno, who uttered all the sentences, including blatantly false ones. For this reason, he could be considered unreliable as a speaker. As Grodner and Sedivy (2011) have shown, when the speaker is presented as unreliable, adult participants do not interpret restrictive modifiers contrastively because they are aware of the fact that the speaker consistently produces overinformative utterances. We might then hypothesize that our participants—possibly also the younger ones—realized that Bruno was not reliable; thus, they decided to sanction the more harmful violations, those that effectively led to communication failures, and overlooked those that were considered to be more innocuous for the purpose of understanding what has happened, which, ultimately, was what they were asked to do.

In the end, we show that if you are asked what you have eaten, and you ate a kiwifruit, the answer “a fruit with a hairy brown skin and a green flesh,” albeit long and weird, is preferable compared to “a banana” (false answer), or “food” (underinformative), or “something with the spoon” (irrelevant).

## Data Availability Statement

The raw data supporting the conclusions of this article will be made available by the authors, without undue reservation.

## Ethics Statement

The studies involving human participants were reviewed and approved by the ethical committee of the University of Milano-Bicocca. Written informed consent to participate in this study was provided by the participants’ legal guardian/next of kin.

## Author Contributions

FP conceived the experimental question, developed the tasks, recruited the children, and supervised the testing. FF performed the statistical analyses. FP drafted the manuscript, which was critically revised by FF. Both authors contributed to the article and approved the submitted version.

## Conflict of Interest

The authors declare that the research was conducted in the absence of any commercial or financial relationships that could be construed as a potential conflict of interest.
