# Supplementary material for: Children’s and Adults’ Sensitivity to Gricean Maxims and to the Maximize Presupposition Principle
Source: Front Psychol. 2021 Mar 3;12:624628. doi: 10.3389/fpsyg.2021.624628 (PMC7966462; doi:10.3389/fpsyg.2021.624628)
Supplement: Supplementary file 1 [file Data_Sheet_1.docx]

**APPENDIX**

Tables A-D. Series of models with type of Medal as the dependent variable, Violation and age Group as independent variables, and subjects and items as random intercepts. Each table reports the output of the model in which a different level of Violation is set as the reference level. In all models, primary-school children is set as the reference level for Group.

Tables E-F-G. Series of models with type of Medal as the dependent variable and Submaxim as independent variables, and subjects and items as random intercepts. Each table reports the output of the model in which a different group is considered. In all models, Be brief is set as the reference level for Submaxim.

**Table A.** Reference level for Violation: Manner.

|  | Estimate | Std. Err. | z-value | p-value |
| --- | --- | --- | --- | --- |
| Violation (MaxPres) | 1.581 | .472 | 3.352 | .001 |
| Violation (Quantity) | -1.467 | .374 | -3.921 | < .001 |
| Violation (Relation) | -1.592 | .458 | -3.477 | .001 |
| Group (Adult) | -.170 | .287 | -.589 | .556 |
| Group (Pre-school) | .630 | .284 | 2.215 | .027 |
| Violation (MaxPres):Group (Adult) | -2.033 | .357 | -5.689 | <. 001 |
| Violation (Quantity):Group (Adult) | -.237 | 0.271 | -.875 | .381 |
| Violation (Relation):Group (Adult) | -.425 | .332 | -1.281 | .200 |
| Violation (MaxPres):Group (Pre-school) | .020 | .420 | .047 | .962 |
| Violation (Quantity):Group(Pre-school) | .3241 | .280 | 1.157 | .247 |
| Violation (Relation):Group(Pre-school) | .750 | .345 | 2.176 | .030 |

**Table B.** Reference level for Violation: Maximize Presupposition.

|  | Estimate | Std. Err. | z-value | p-value |
| --- | --- | --- | --- | --- |
| Violation (Manner) | -1.581 | .472 | -3.352 | < .001 |
| Violation (Quantity) | -3.048 | .474 | -6.436 | < .001 |
| Violation (Relation) | -3.174 | .543 | -5.852 | < .001 |
| Group (Adult) | -2.202 | .365 | -6.033 | < .001 |
| Group (Pre-school) | .650 | .417 | 1.557 | .119 |
| Violation (Manner):Group (Adult) | 2.033 | .357 | 5.689 | < .001 |
| Violation (Quantity):Group (Adult) | 1.800 | .352 | 5.105 | < .001 |
| Violation (Relation):Group (Adult) | 1.608 | .400 | 4.018 | < .001 |
| Violation (Manner):Group (Pre-school) | -.020 | .420 | -.047 | .962 |
| Violation (Quantity):Group (Pre-school) | .304 | .413 | .736 | .462 |
| Violation (Relation):Group (Pre-school) | .730 | .460 | 1.589 | .112 |

**Table C.** Reference level for Violation: Quantity I.

|  | Estimate | Std. Err. | z-value | p-value |
| --- | --- | --- | --- | --- |
| Violation (MaxPres) | 3.048 | .477 | 6.436 | < .001 |
| Violation (Manner) | 1.467 | 0.374 | 3.921 | < .001 |
| Violation (Relation) | -.126 | .456 | -.276 | .783 |
| Group (Adult) | -.406 | .280 | -1.449 | .147 |
| Group (Pre-school) | .954 | .270 | 3.532 | < .001 |
| Violation (MaxPres):Group (Adult) | -1.800 | .352 | -5.105 | < .001 |
| Violation (Manner):Group (Adult) | .238 | .271 | .875 | .381 |
| Violation (Relation):Group (Adult) | -.188 | .326 | -.577 | .564 |
| Violation (MaxPres):Group (Pre-school) | -.304 | .413 | -.736 | .462 |
| Violation (Manner):Group (Pre-school) | -.324 | .280 | -1.157 | .247 |
| Violation (Relation):Group (Pre-school) | .426 | .333 | 1.280 | .201 |

**Table D.** Reference level for Violation: Relation.

|  | Estimate | Std. Err. | z-value | p-value |
| --- | --- | --- | --- | --- |
| Violation (Quantity) | .126 | .455 | .276 | .783 |
| Violation (Max Pres) | 3.174 | .542 | 5.851 | < .001 |
| Violation (Manner) | 1.592 | .458 | 3.477 | .001 |
| Group (Adult) | -.594 | .340 | -1.750 | .080 |
| Group (Pre-school) | 1.380 | .337 | 4.095 | < .001 |
| Violation (Quantity):Group (Adult) | .188 | .326 | 0.577 | .564 |
| Violation (Max Pres):Group (Adult) | -1.608 | .400 | -4.018 | < .001 |
| Violation (Manner):Group (Adult) | .425 | .332 | 1.281 | .200 |
| Violation (Quantity):Group (Pre-school) | -.426 | .333 | -1.280 | .201 |
| Violation (Max Pres):Group (Pre-school) | -.730 | .460 | -1.589 | .112 |
| Violation (Manner):Group (Pre-school) | -.750 | .345 | -2.176 | .030 |

**Table E.** Group: adults.

|  | Estimate | Std. Err. | z-value | p-value |
| --- | --- | --- | --- | --- |
| Sub.Max (Quantity) | -2.307 | .677 | -3.408 | .001 |
| Sub.Max (Be orderly) | 1.177 | .732 | 1.608 | .108 |
| Sub.Max (Max Pres) | -.171 | .741 | -.230 | .818 |
| Sub.Max (Relation) | -2.868 | .784 | -3.658 | < .001 |

**Table F.** Group: primary-school children.

|  | Estimate | Std. Err. | z-value | p-value |
| --- | --- | --- | --- | --- |
| Sub.Max (Quantity) | -1.467 | .451 | -3.251 | .001 |
| Sub.Max (Be orderly) | .015 | .522 | .029 | .977 |
| Sub.Max (Max Pres) | 1.541 | .532 | 2.899 | .004 |
| Sub.Max (Relation) | -1.603 | .522 | -3.074 | .002 |

**Table G.** Group: pre-school children.

|  | Estimate | Std. Err. | z-value | p-value |
| --- | --- | --- | --- | --- |
| Sub.Max (Quantity) | -.654 | .578 | -1.132 | .258 |
| Sub.Max (Be orderly) | .674 | .682 | .988 | .323 |
| Sub.Max (Max. Pres) | 1.600 | .704 | 2.272 | .0231 |
| Sub.Max (Relation) | -.413 | .668 | -.618 | .536 |
